# Prognostic Value of Modified Model for End-Stage Liver Disease Score in Patients Undergoing Isolated Tricuspid Valve Replacement

**DOI:** 10.3389/fcvm.2022.932142

**Published:** 2022-07-01

**Authors:** Hongjie Xu, He Wang, Shisong Chen, Qian Chen, Tianyu Xu, Zhiyun Xu, Yangyong Sun

**Affiliations:** ^1^Department of Cardiovascular Surgery, Changhai Hospital, Naval Medical University, Shanghai, China; ^2^Department of Cardiothoracic Surgery, Affiliated People’s Hospital of Jiangsu University, Zhenjiang, China

**Keywords:** ITVR, TR, hepatorenal dysfunction, modified MELD score, in-hospital prognosis

## Abstract

**Objective:**

Though the prognostic value of the model for end-stage liver disease (MELD) score in tricuspid surgery was confirmed, the unstable international normalized ratio (INR) may affect the evaluation effectiveness of the MELD score for isolated tricuspid valve replacement (ITVR). The aim of the study was to assess the prognostic value of modified MELD for ITVR.

**Methods and Results:**

A total of 152 patients who underwent ITVR were evaluated. The adverse outcome was defined as in-hospital mortality after surgery. The receiver operating characteristic (ROC) curve analysis demonstrated that a modified MELD score with albumin replacing INR (MELD-albumin) score presented well prognostic value [area under the curve (AUC) = 0.731, *p* = 0.006] for in-hospital mortality. Through Cox regression and further interval validation, the MELD-albumin score was identified as an independent predictor for in-hospital mortality. The optimal cutoff value of MELD-albumin was identified as 8.58 through maximally selected log-rank statistics. In addition, restricted cubic spline analysis demonstrated the linear inverse relationship between MELD-albumin and hazard ratio (HR) for in-hospital mortality. Kaplan–Meier analysis illustrated that in-hospital mortality was increased significantly in the high MELD-albumin (MELD-albumin ≥8.58) group than in the low MELD-albumin group (MELD-albumin <8.58; *p* < 0.001). Furthermore, high MELD-albumin was associated with lower body mass index (BMI), the incidence of lower extremities edema and moderate drinking history, and the MELD-albumin score was correlated with the value of aspartate transaminase (AST), alanine transaminase (ALT), and albumin. Furthermore, the incidence of renal failure (*p* = 0.003) and pulmonary infection (*p* = 0.042) was increased significantly in the high MELD-albumin group.

**Conclusion:**

The MELD-albumin score could provide prognostic value for ITVR. In addition, the MELD-albumin score was useful in risk stratification and patient selection for patients with tricuspid regurgitation (TR) prior to ITVR.

## Introduction

Tricuspid regurgitation (TR) affects more than 1.6 million people in the United States, but it was remarkably undertreated when compared with left valvular disease (1). The main therapy for patients with TR is conservative management to alleviate symptoms, whereas isolated tricuspid valve replacement (ITVR) was rarely performed (2). To date, the tricuspid valve maneuvers, such as ITVR and tricuspid repair, were recommended to perform only when TR was severe with concomitant right-sided heart failure, presenting with a dismal prognosis. Compared with people prone to tricuspid repair, the population prior to ITVR has higher opportunities for reoccurrence of TR and more complex conditions. Thus, it was critical to provide an accurate evaluation for the prognosis of ITVR to support clinical decisions (3). However, there were few attempts to establish a scoring system only for the outcomes of ITVR (4). Additionally, cardiorenal and cardiohepatic syndromes, as the common complications of heart failure that affect TR prognosis, were hardly enrolled in the prognostic model of ITVR (5).

The model for end-stage liver disease (MELD) score, calculated by formula involved with the value of serum total bilirubin, creatinine, and international normalized ratio (INR), focuses more on the systemic hepatorenal-cardiac interaction (6). Moreover, the MELD score was expected as a promising predictive tool the for prognosis of TR surgery (7), but a previous study presented that the MELD score performed similarly to the conventional cardiovascular scoring system (8). However, the unstable INR resulting from the oral anticoagulation treatment was common in patients prior to ITVR surgery, which may affect the predictive accuracy of the MELD score. Recently, modified MELD scores, excluding INR (MELD-XI) or substituting INR with albumin (MELD-albumin), were developed to optimize the traditional MELD scores. Moreover, the prognostic value of modified MELD scores has been demonstrated for transcatheter or surgical tricuspid repair (9, 10). However, the prognostic value of both MELD and modified MELD scores in patients who underwent ITVR was unknown. It was imperative to investigate the prognostic value of modified MELD scores for ITVR to provide suggestions for risk stratification and perioperative management. Thus, the aim of this study was to evaluate the predictive ability of modified MELD scores for adverse outcomes in patients with ITVR.

## Materials and Methods

### Study Population

From 2005 to 2020, the data of patients who underwent ITVR at Naval Medical University, Changhai Hospital were collected. After patients with previous hepatogenic and nephrogenic diseases or missed data were excluded, the clinical data of 152 patients were enrolled in the study. The adverse outcome was defined as in-hospital mortality after surgery. The study was approved by the Ethics Committee of Changhai Hospital on the human research. Moreover, the clinical data were obtained from the electronic medical record with written informed consent from patients.

### Clinical Parameters

In addition, basic information, such as age, sex, and body mass index (BMI), and conventional cardiovascular risk factors, such as the history of diabetes, hypertension, stroke, smoking, moderate drinking, previous cardiac surgery, and pacemaker implantation, were documented. Notably, smoking was defined as having smoked at least one cigarette per day for 1 year (11). Previous moderate drinking was defined as the history of one or two drinks per day (12). New York Heart Association classification was recorded as class III/IV or others. The status of atrial fibrillation was collected from medical records or electrocardiogram. The severity of TR and left ventricular ejection fraction (LVEF) was from echocardiographic data, and the occurrence of liver congestion was identified by abdominal ultrasonography. The cardiopulmonary bypass time (CPBT) and prosthesis size were obtained from surgical records. The incidence of postoperative renal failure was diagnosed based on Acute Kidney Injury Network (AKIN) criteria (13). The diagnosis of postoperative pulmonary infection was based on the computed tomography (CT) and bacterial culture of sputum. In addition, the diagnosis of reoperation for bleeding was according to the indicator that the thoracic drainage volume was more than 200 ml/h for 3 consecutive hours (14). The laboratory parameters were collected from the recent blood analysis before surgery. In addition, the values of alanine transaminase (ALT) and aspartate transaminase (AST) were also obtained to reflect hepatic function. The estimated glomerular filtration rate (eGFR) was calculated with creatinine according to the previous study (15). MELD score, MELD-XI score, and MELD-albumin score were calculated as followed (9):


MELDscore=11.2×In(INR)+3.78×In(totalbilirubin)+9.57×In(creatinine)+6.43.

MELD-XIscore=5.11×In(totalbilirubin)+11.76×In(creatinine)+9.44.

MELD-albuminscore=11.2×In(1)+3.78×In(totalbilirubin)+9.57×In(creatinine)+6.43,(thisformulawasperformedwhenthevalueofalbuminwas≥4.1g/dl).

MELD-albuminscore=11.2×In[1+(4.1-albumin)]+3.78×In(totalbilirubin)+9.57×In(creatinine)+6.43,(thisformulawasperformedwhenthevalueofalbuminwas4.1g/dl).

### Statistical Analysis

Software of R 4.1.1 (R Foundation for Statistical Computing, Vienna, Austria) and SPSS 20 were used for the analysis. For continuous variables, normally distributed variables were depicted as mean ± standard deviation (SD) and compared by *t*-test, and non-normally distributed variables were presented as medians with an interquartile and compared by Mann–Whitney *U* test. Whereas, categorical variables were presented as frequency (percentage) and compared by chi-square test or Fisher’s exact test. The receiver operating characteristic (ROC) curves of hepatorenal biomarkers were depicted by using logistic analysis for predicting in-hospital mortality. Moreover, the net classification index (NRI) was calculated for comparison of ROC curves between MELD-albumin and others. Cox proportional hazard regression was performed to identify the predictors for in-hospital mortality, and variables with *p* < 0.05 in the univariate analysis were incorporated into a multivariable regression model. Furthermore, cox proportional hazard regression with the method of bootstrapping 1,000 times was performed for interval validation. Furthermore, the distribution of the MELD-albumin score was exhibited and the optimal cutoff value of the MELD-albumin score was identified through maximally selected log-rank statistics. After the linear inverse relationship between MELD-albumin and HR for in-hospital mortality was identified by restricted cubic spline analysis, the patients were divided into groups according to the optimal cutoff value of MELD-albumin. Kaplan–Meier analysis was used to evaluate in-hospital survival, with the survival function using a log-rank test. Moreover, factors associated with MELD-albumin were identified by logistic regression. In the multivariable analyses, covariates were included that showed significance (*p* < 0.05) in the univariable analysis. The correlation between MELD-albumin score and continuous variables was performed by the mantel test. The value of *p* < 0.05 was deemed as statistical significance.

## Results

### Baseline Clinical Characteristics of Study Population

In the study, the baseline clinical characteristics of 152 patients who underwent ITVR were delineated. Moreover, there were 87 women (57.2%) and 65 men (42.8%), with average age of 55.1 ± 12.0 years. Totally, 13 patients died in hospital after surgery and the in-hospital mortality was presented as 8.6%. Regarding the etiology of the patients leading to the ITVR, 77.6% of the patients underwent previous cardiac surgery with the reoccurrence of TR, 3.3% of the patients suffered from endocarditis, and 5.9% of the patients suffered from congenital heart disease. The patients in death group showed deteriorated hepatorenal function, presented as high MELD score (11.4 ± 4.2 vs. 13.7 ± 6.47, *p* = 0.078), MELD-XI score [9.4 (9.4, 9.4) vs. 9.4 (9.4–15.3), *p* = 0.001], and MELD-albumin score [7.3 (6.4, 9.4) vs. 10.0 (8.6, 13.6), *p* = 0.004]. Other clinical parameters are presented in Table 1.

**TABLE 1:** Baseline characteristics of patients.

Variables	Overall (*n* = 152)	Survival (*n* = 139)	Death (*n* = 13)	*P* Value
Age, years	55.1 ± 12.0	54.7 ± 11.7	59.8 ± 14.5	0.144
Female	87 (57.2)	82 (59.0)	5 (5.7)	0.240
BMI	22.1 ± 3.1	22.2 ± 3.2	21.8 ± 2.5	0.716
Smoking	13 (8.6)	10 (7.2)	3 (23.1)	0.085
Moderate drinking	7 (4.6)	5 (3.6)	2 (15.4)	0.111
Hypertension	17 (11.2)	15 (10.8)	2 (15.4)	0.641
Stroke	7 (4.6)	6 (4.3)	1 (7.7)	0.472
Diabetes	13 (8.6)	11 (7.9)	2 (15.4)	0.307
NYHA class III/IV	96 (63.2)	83 (59.7)	13 (100)	0.002*
Atrial fibrillation	92 (60.5)	85 (61.2)	7 (53.8)	0.768
Etiology for ITVR				0.222
Previous cardiac surgery	118 (77.6)	110 (79.1)	8 (61.5)	
Infective endocarditis	5 (3.3)	4 (2.9)	1 (7.7)	
Congenital heart diseases	9 (5.9)	8 (5.8)	1 (7.7)	
Others	20 (13.2)	17 (12.2)	3 (23.1)	
Pacemaker implantation	11 (7.2)	9 (6.5)	2 (15.4)	0.239
Lower extremities edema	77 (50.7)	68 (48.9)	9 (69.2)	0.246
Severe TR insufficiency	145 (95.4)	133 (95.7)	12 (92.3)	0.472
LVEF	61.8 ± 8.4	62.1 ± 8.2	59.4 ± 10.4	0.269
Liver congestion	68 (44.7)	62 (44.6)	6 (46.2)	1.000
Pericardial effusion	5 (3.3)	5 (3.6)	0 (0)	1.000
Pleural effusion	25 (16.4)	21 (15.1)	4 (30.8)	0.230
eGFR	88.5 ± 79.3	90.5 ± 82.1	66.9 ± 31.7	0.306
Total bilirubin	23.1 ± 18.3	23.0 ± 18.7	24.3 ± 13.5	0.813
INR	1.7 ± 0.7	1.7 ± 0.7	1.7 ± 0.7	0.939
Albumin	40.1 ± 4.1	40.2 ± 4.4	39.0 ± 6.0	0.366
AST	26 (22, 32)	26 (22, 32)	26 (23, 33.5)	0.707
ALT	18 (14, 25)	18 (14, 25)	15 (12.5, 25.5)	0.484
MELD score	11.6 ± 4.5	11.4 ± 4.2	13.7 ± 6.47	0.078
MELD-XI score	9.4 (9.4, 9.4)	9.4 (9.4, 9.4)	9.4 (9.4, 15.3)	0.001*
MELD-albumin score	7.5 (6.4, 10.1)	7.3 (6.4, 9.4)	10.0 (8.6, 13.6)	0.004*
CPBT	64.5 (49, 90)	63 (48, 88)	84 (54, 119)	0.121
Prosthesis size	30.2 ± 1.2	30.2 ± 1.2	30.1 ± 1.6	0.762

^a^BMI, body mass index; NYHA, New York Heart Association classification; TR, tricuspid regurgitation; LVEF, left ventricular ejection fraction; INR, international normalized ratio; eGFR, estimated glomerular filtration rate; AST, aspartate transaminase; ALT, alanine transaminase; MELD, Model for End-stage Liver Disease; MELD-XI, Model for End-stage Liver Disease excluding international normalized ratio; MELD-albumin, Model for End-stage Liver Disease with albumin replacing international normalized ratio. The value of P < 0.05 was noted as bold value with *.

### Prognostic Value of Modified Model for End-Stage Liver Disease Scores for In-Hospital Mortality

**FIGURE 1:**
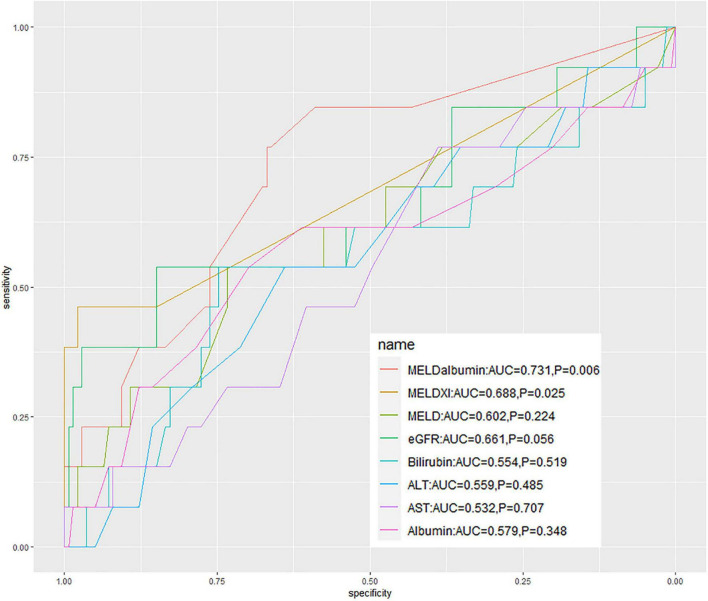
The receiver operating characteristic curves of the hepatorenal biomarkers to determine the accuracy to predict the in-hospital mortality following ITVR. MELD, a model for end-stage liver disease; MELD-albumin, model for end-stage liver disease with albumin replacing international normalized ratio; MELD-XI, model for end-stage liver disease excluding international normalized ratio; eGFR, estimated glomerular filtration rate; ALT, alanine transaminase; AST, aspartate transaminase; AUC, area under the curve.

Subsequently, the ROC curves were established to determine the accuracy of hepatorenal biomarkers to predict in-hospital mortality following ITVR. Modified MELD scores, such as MELD-albumin [area under the curve (AUC) = 0.731, *p* = 0.006] and MELD-XI (AUC = 0.688, *p* = 0.025), presented better predictive ability (Supplementary Table 1). Nevertheless, the prognostic values of MELD score, albumin, eGFR, total bilirubin, AST, and ALT were low statistically (Figure 1). Thus, among the factors reflecting hepatorenal function, MELD-albumin was selected for further analysis based on the value of AUC. Furthermore, Cox regression analysis was performed to identify the predictors of in-hospital mortality after surgery (Table 2). Through univariate analysis, the previous moderate drinking history, AST, ALT, cardiopulmonary bypass time (CPBT), and MELD-albumin score were significantly associated with in-hospital mortality after ITVR. Moreover, MELD score was demonstrated as no statistical significance for in-hospital mortality (*p* = 0.076). Then multivariable Cox regression analysis was performed involving significant variables in univariate analysis. It was demonstrated that MELD-albumin [hazard ratio (HR) 1.233, 95% CI 1.060–1.435, *p* = 0.007] remained a significant prognostic value for in-hospital mortality after ITVR. In addition, Cox regression analysis with bootstrapping of 1,000 times further validated the result (Supplementary Table 2).

**TABLE 2:** Cox regression analysis for perioperative characteristics predictive of in-hospital mortality.

Variables	Univariate		Multivariable (MELD-albumin)	
		
	HR (95% CI)	*P* Value	HR (95% CI)	*P* Value
Age, years	1.038 (0.988–1.091)	0.136		
Female	0.4527 (0.148–1.384)	0.165		
BMI	0.966 (0.809–1.153)	0.699		
Smoking	3.358 (0.924–12.21)	0.066		
Moderate drinking	6.395 (1.411–28.98)	0.016*	2.993 (0.339–26.457)	0.324
Hypertension	1.462 (0.3241–6.598)	0.621		
Stroke	1.758 (0.229–13.52)	0.588		
Diabetes	2.06 (0.457–9.294)	0.347		
NYHA class III/IV	0.023 (0.000–2.345)	0.110		
Atrial fibrillation	0.754 (0.254–2.245)	0.612		
Previous cardiac surgery	0.444 (0.145–1.358)	0.155		
Pacemaker implantation	2.649 (0.587–11.96)	0.205		
Lower extremities edema	2.248 (0.692–7.301)	0.178		
Severe TR insufficiency	0.548 (0.0713–4.217)	0.564		
LVEF	0.967 (0.907–1.031)	0.310		
Liver congestion	1.071 (0.360–3.188)	0.901		
Pericardial effusion	0.047 (0.000–28306.0)	0.653		
Pleural effusion	2.3 (0.708–7.47)	0.166		
AST	1.001 (1.001–1.002)	0.002*	1.022 (0.975–1.072)	0.361
ALT	1.003 (1.001–1.005)	0.003*	0.955 (0.866–1.053)	0.357
MELD score	1.102 (0.990–1.228)	0.076		
MELD-albumin score	1.231 (1.107–1.368)	<0.001*	1.233 (1.060–1.435)	0.007*
CPBT	1.006 (1.003–1.009)	<0.001*	1.008 (1.004–1.011)	<0.001*
Prosthesis size	0.894 (0.588–1.361)	0.601		

^a^BMI, body mass index; NYHA, New York Heart Association classification; TR, tricuspid regurgitation; LVEF, left ventricular ejection fraction; INR, international normalized ratio; AST, aspartate transaminase; ALT, alanine transaminase; MELD, Model for End-stage Liver Disease; MELD-albumin, Model for End-stage Liver Disease with albumin replacing international normalized ratio; CPBT, cardiopulmonary bypass time. The value of P < 0.05 was noted as bold value with *.

### In-Hospital Survival Analysis

**FIGURE 2:**
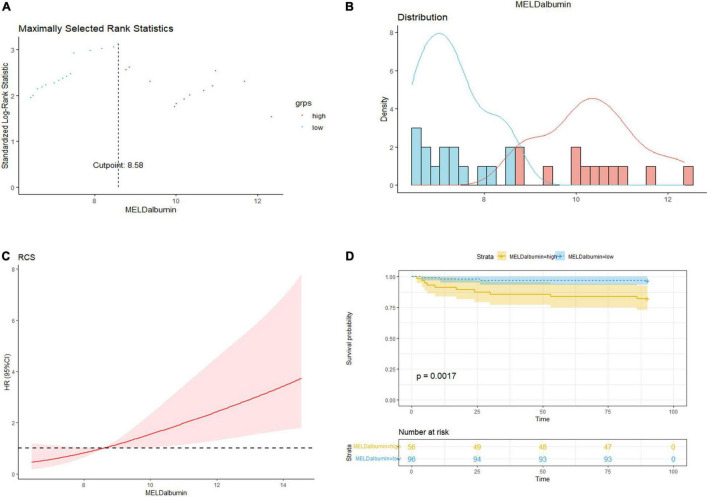
Prognostic value of MELD-albumin score for in-hospital mortality. **(A)** The maximally selected log-rank statistics of MELD-albumin for survival indicated that optimal cutoff value of MELD-albumin was 8.58. **(B)** The distribution of MELD-albumin in patients of low MELD-albumin group (blue) and high MELD-albumin group (red) respectively based on survival. **(C)** Restricted cubic spline analysis for the relationship between MELD-albumin score and hazard ratio for in-hospital mortality in patients with the cutoff value of 8.58. **(D)** Kaplan–Meier curve analysis for the in-hospital mortality between high MELD-albumin (≥8.58) and low MELD-albumin (<8.58) groups.

Then the data were analyzed through maximally selected log-rank statistics, and the optimal cutoff value of MELD-albumin was identified as 8.58 (Figure 2A). Then, the patients were divided into two groups based on the optimal value of MELD-albumin, which presented as high MELD-albumin group (MELD-albumin ≥8.58) and low MELD-albumin group (MELD-albumin <8.58). Moreover, the distribution of MELD-albumin is exhibited in Figure 2B, the average values of MELD-albumin scores in the high MELD-albumin group and the low MELD-albumin group are 11.6 ± 3.1 and 6.9 ± 0.7, respectively. In addition, restricted cubic spline analysis showed a relatively linear inverse relationship between MELD-albumin and HR for in-hospital mortality in patients with the cutoff value of 8.58 (Figure 2C). In addition, the results of the Kaplan–Meier analysis indicated that in-hospital mortality following ITVR was higher in high MELD-albumin group than low MELD-albumin group (*p* = 0.0017; Figure 2D), which further demonstrated the prognostic value of MELD-albumin. In addition, cutoff values from previous studies were selected for sensitivity analysis (9, 16). The Kaplan–Meier analysis showed that the probability of in-hospital mortality was significantly higher in patients with high MELD-albumin score based on the cutoff values from other studies (Supplementary Figure 1).

### Variables Associated With Deteriorated Hepatorenal Function by Model for End-Stage Liver Disease-Albumin Score

**FIGURE 3:**
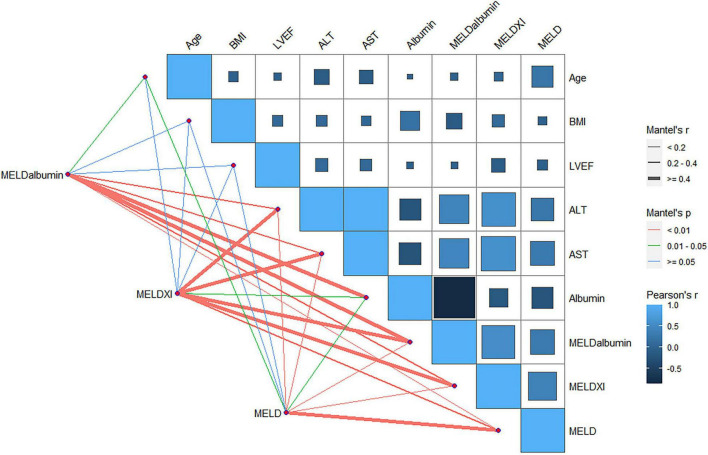
The correlation between MELD, MELD-XI or MELD-albumin, and continuous variables in patients following ITVR by mantle test. MELD, model for end-stage liver disease; MELD-albumin, model for end-stage liver disease with albumin replacing international normalized ratio; MELD-XI, model for end-stage liver disease excluding international normalized ratio; BMI, body mass index; ALT, alanine transaminase; AST, aspartate transaminase.

Clinical parameters contributing to the deteriorated hepatorenal function by MELD-albumin were identified. As shown in Supplementary Table 3, after univariate logistic analysis, factors associated with higher MELD-albumin scores are delineated. Additionally, the results through multivariable logistic analysis identified that lower BMI [odds ratio (OR) 0.866 95% CI 0.768–0.977 *p* = 0.019], the incidence of moderate drinking history (OR 15.879 95% CI 1.759–143.32 *p* = 0.014), and lower extremities edema (OR 2.434 95% CI 1.190–4.976 *p* = 0.015) were independent predictors of higher MELD-albumin score (Table 3). Furthermore, the correlation between continuous variables and MELD-derived scores was analyzed (Figure 3). MELD score, MELD-XI score, and MELD-albumin score were all positively correlated with the value of AST and ALT but negatively correlated with serum albumin. Different from the MELD score and MELD-XI score, the MELD-albumin score was related to serum albumin more tightly than the values of AST and ALT, indicating the different emphasis of the MELD-albumin score.

**TABLE 3:** Variables associated with elevating higher MELD-albumin score.

Variables	OR (95% CI)	*P* Value
BMI	0.866 (0.768–0.977)	0.019*
Moderate drinking	15.879 (1.759–143.32)	0.014*
Lower extremities edema	2.434 (1.190–4.976)	0.015*

^a^BMI, body mass index. The value of P < 0.05 was noted as bold value with *.

### Postoperative Complications for Deteriorated Hepatorenal Function by Model for End-Stage Liver Disease-Albumin Score

Furthermore, the incidence rates of postoperative complications following ITVR were compared between different MELD-albumin groups, which are shown in Table 4. After surgery, there was no statistical significance in the incidence of reoperation for bleeding, pericardial effusion drainage and pleural effusion drainage, and pleural fluid 24 h after surgery between two groups. Nevertheless, the occurrence of renal failure (2.1 vs. 15.8%, *p* = 0.003) and pulmonary infection (5.3 vs. 15.8%, *p* = 0.042) was more common in high MELD-albumin group. Moreover, patients in high MELD-albumin group presented longer time stay of hospital (18.3 ± 5.9 vs. 24.4 ± 16.6, *p* = 0.037) and intensive care unit (3.4 ± 3.6 vs. 8.1 ± 15.3, *p* = 0.025).

**TABLE 4:** Postoperative outcomes for different MELD-albumin groups.

Variables	MELD-albumi*n* <8.58 (*n* = 95)	MELD-albumin ≥8.58 (*n* = 57)	Overall (*n* = 152)	*P* Value
Reoperation for bleeding	4 (4.2)	2 (3.5)	6 (3.9)	1.000
Renal failure	2 (2.1)	9 (15.8)	11 (7.2)	0.003*
Pulmonary infection	5 (5.3)	9 (15.8)	14 (8.2)	0.042*
Pleural fluid in 24 h	453.8 ± 394.6	487.6 ± 519.2	466.5 ± 444.1	0.651
Pericardial effusion drainage	7 (7.4)	9 (15.8)	16 (10.5)	0.170
Pleural effusion drainage	64 (67.4)	43 (75.4)	107 (70.4)	0.360
Hospital length of stay	17 (14, 21)	19 (15, 30.5)	18 (14, 23)	0.037*
ICU length of stay	2 (2, 4)	3 (2, 5.5)	3 (2, 4)	0.025*
Death	3 (3.2)	10 (17.5)	13 (8.6)	0.003*

^a^MELD-albumin, Model for End-stage Liver Disease with albumin replacing international normalized ratio; ICU, intensive care unit. The value of P < 0.05 was noted as bold value with *.

## Discussion

The results of our study indicated that patients with TR who underwent ITVR were common with deteriorated hepatorenal function, presented as both high MELD scores and modified MELD scores. In addition, the MELD-albumin score, an objective biomarker reflecting hepatorenal function, could provide strong predictive value for adverse outcomes of patients following ITVR in hospital. Nevertheless, the prognostic value of MELD score was weakened in ITVR. In addition, it was found that MELD-albumin score was closely related to the BMI and albumin, apart from deteriorated hepatorenal dysfunction by AST and ALT. Thus, the present study provided evidence for the appliance of MELD-albumin score to predict adverse outcomes in patients with TR who underwent ITVR after surgery in hospital.

According to the guidelines, the procedure of ITVR should be performed in patients with TR when TR repair is not technically feasible or TR repair is assessed with high risk of reoccurrence (17). Thus, the conditions of patients who underwent ITVR seem more complicated. When assessing the risk of ITVR, extracardiac organ damage is closely related to and has strong impact on the prognosis. Among them, cardiohepatic and cardiorenal syndromes, occurring in the setting of severe venous congestion, are the pathological damages of TR (18, 19). In addition, previous study demonstrated that patients who underwent isolated TR surgery were prone to poor prognosis with higher prevalence of chronic kidney and liver dysfunction (20). Thus, kidney- and liver-associated biomarkers have been recommended to predict poor outcomes of TR surgery. Thus, suitable biomarkers accounting for hepatic and renal function were reasonable to be involved in the risk evaluation of ITVR. MELD scores, as combined assessment of liver and kidney functions, could also reflect hepatorenal-cardiac interaction in patients with TR.

The model for end-stage liver disease score was initially developed for risk stratification in liver cirrhosis, which provided an assessment for hepatorenal dysfunction. Recently, accumulative pieces of evidence indicated that the MELD score may be useful in assisting the risk evaluation for TR surgery (21, 22). Moreover, studies suggested that mortality of TR surgery was increased incrementally with worsening MELD score, but the procedure of tricuspid valve replacement accounted for only 7.1% proportion in the study (23). The data of 152 patients who underwent ITVR in our study suggested that the significance of the MELD score for in-hospital mortality in ITVR was low statistically. Regardless of bias in the study, the majority of patients enrolled in our study were presented with an unstable value of INR that was caused by oral anticoagulation treatment. Even if the INR was rectified by Vit K1 treatment or reduced anticoagulation dose, the prognostic value of the MELD score was constricted or was unstable under this condition. To optimize the MELD score, INR was excluded (MELD-XI) or replaced by albumin (MELD-albumin) to accurately reflect hepatopathy in patients prescribed with anticoagulation (24, 25). Moreover, it seemed that a modified MELD score could provide a strong ability to evaluate in-hospital mortality and morbidity following ITVR. So far, previous studies did not distinguish whether the patients with TR received anticoagulation therapy. Thus, whether modified MELD scores performed better in patients without anticoagulation therapy required further investigation.

It was noteworthy that modified MELD scores had a more accurate prognostic value for adverse outcomes of ITVR than other hepatorenal biomarkers enrolled in the study. Apart from the biomarkers of hepatorenal function, the serum albumin is also a stable protein that reflects the regulatory thrombo-inflammatory status and physical nutritional level. Evidence from studies suggested that serum albumin functioned as an antioxidant regulator in cellular inflammatory signaling, while decreased synthesis and increased catabolism of serum albumin were related to inflammation (26). Additionally, serum albumin was particularly noteworthy as an indicator for frailty and cachexia in patients with TR. Consistently, the result of our study showed that the MELD-albumin score was significantly associated with low BMI, reflecting the nutritional status by albumin. Accumulative pieces of evidence have demonstrated that systemic inflammation and frailty were of utmost importance to adverse outcomes in patients with TR (27). Therefore, it was reasonable to prospect that albumin promotes the predictive value for patients with TR following ITVR. Nevertheless, the value of serum albumin may be affected by current nutritional condition and inflammatory status, which may account for factors other than TR etiology. Therefore, more studies involving data of patients from multiple centers were warranted to clarify the prognostic value of MELD-albumin in ITVR.

The development of the risk evaluation system for TR is historically challenging. Traditional scoring systems, such as the European System for Cardiac Operative Risk Evaluation (EuroSCORE) and the Society of Thoracic Surgeons (STS) score, were applied to TR risk assessment in several studies, but they exhibited limited role and were not specific for isolated TR surgery. Therefore, accumulative attempts were constantly performed to establish an accurate scoring system for evaluating the surgical risk of patients with TR. Recently, a clinical risk assessment tool was developed to help in predicting mortality and morbidity of tricuspid valve surgery, but the hepatorenal information was not involved and 86% of patients in the study underwent tricuspid repair (28). Furthermore, TRI-SCORE, a more comprehensive score for in-hospital mortality prediction after isolated tricuspid surgery, was established and required further validation in different population subsets (29). Moreover, echocardiographic parameters for the assessment of right ventricular systolic function involved in the TRI-SCORE were subjected to intrinsic limitations (29). Though our study confirmed the strong predictive value of MELD-albumin for in-hospital mortality and morbidity in ITVR, the MELD-albumin mainly reflects the effect of the hepatorenal function. Thus, a comparison of prognostic value between MELD-albumin and TRI-SCORE in patients following ITVR and whether MELD-albumin could improve the predictive ability of TRI-SCORE merited further investigation.

Taken together, our study provided strong evidence that MELD-albumin as objective index derived from serum biomarkers could provide effective prognostic value for in-hospital mortality and morbidity in patients with TR following ITVR. Furthermore, current guidelines recommend tricuspid valvular surgery before the multi-organ damage (30). Armed with this knowledge, we recommended that MELD-albumin should be considered in the preoperative evaluation scoring system prior to ITVR for risk stratification and patient selection. Furthermore, the prognostic value of the modified MELD score for transcatheter tricuspid repair has been confirmed by a previous study (10). For patients with high MELD-albumin scores, modified MELD scores could be a useful tool to select patients who might be better suitable for transcatheter tricuspid repair.

To our knowledge, even though this study delineated the prognostic value of MELD-albumin score in patients with TR following ITVR for the first time, there were several limitations. Firstly, the study was a retrospective study using a small size of database from one center, which may cause bias and limit statistical validity. Thus, further studies involving multiple centers in different population subsets should be required. In addition, the etiology of patients was complicated, and whether patients were suffered from chronic hepatorenal dysfunction or acute one was not distinguished, which may have an impact on the prognosis. Moreover, the data of long-term follow-up were not included in our study, and whether MELD-albumin could predict the hepatorenal functional recovery after procedure merited further investigation. Additionally, the data on preoperative right ventricular function and postoperative echocardiography were not analyzed, which may constrict the accuracy of the study.

## Conclusion

Our results provide clues to that MELD-albumin, as an easily calculated and objective scoring model, aids in risk stratification and patient selection for patients with TR prior to ITVR.

## Ethics Statement

The studies involving human participants were reviewed and approved by the Ethics Committee of Changhai Hospital. The patients/participants provided their written informed consent to participate in this study.

## Author Contributions

YS and ZX conceived and designed the study. HX, HW, QC, and TX collected the clinical data. HX, HW, and SC performed the data analysis and interpretation. YS, HX, and SC wrote the manuscript. All authors contributed to the article and approved the submitted version.

## Conflict of Interest

The authors declare that the research was conducted in the absence of any commercial or financial relationships that could be construed as a potential conflict of interest.

## Publisher’s Note

All claims expressed in this article are solely those of the authors and do not necessarily represent those of their affiliated organizations, or those of the publisher, the editors and the reviewers. Any product that may be evaluated in this article, or claim that may be made by its manufacturer, is not guaranteed or endorsed by the publisher.

## Supplementary Material

Supplementary Figure 1 Kaplan–Meier curve analysis for the in-hospital mortality with cutoff value of the model for end-stage liver disease (MELD)-albumin from previous studies. **(A)** Kaplan–Meier curve analysis for the in-hospital mortalitywith cutoff value of MELD-albumin as 10.7. **(B)** Kaplan–Meier curve analysis for the in hospitalmortality with cutoff value of MELD-albumin as 7.9.
