# Supplementary material for: Prognostic Value of Modified Model for End-Stage Liver Disease Score in Patients Undergoing Isolated Tricuspid Valve Replacement
Source: Front Cardiovasc Med. 2022 Jul 1;9:932142. doi: 10.3389/fcvm.2022.932142 (PMC9283717; doi:10.3389/fcvm.2022.932142)
Supplement: Supplementary file 1 [file Table_1.docx]

Supplementary Table 1 The comparison of predictive value between MELD-albumin and other indexes

|  | MELDXI | MELD | eGFR | Bilirubin | ALT | AST | Albumin |
| --- | --- | --- | --- | --- | --- | --- | --- |
| NRI | 1.4% | 23.1% | 7.7% | 23.1% | 15.4% | 15.4% | 23.1% |

^a^ NRI, net reclassification index; AST, aspartate transaminase; ALT, alanine transaminase; MELD-albumin, Model for End-stage Liver Disease with albumin replacing international normalized ratio. CPBT, cardiopulmonary bypass time.

Supplementary Table 2 Interval validation of Cox regression analysis with bootstrapping

| Variables | SE | 95%CI | *P* Value |
| --- | --- | --- | --- |
| Moderate drinking | 20.944 | -47.741-3.528 | 0.323 |
| AST | 0.039 | -0.041-0.118 | 0.359 |
| ALT | 0.067 | -0.234-0.029 | 0.371 |
| MELD-albumin score | 0.074 | 0.076-0.372 | **0.002^*^** |
| CPBT | 0.005 | 0.001-0.018 | **0.001^*^** |

^a^ SE, standard error. CI, confidence interval. AST, aspartate transaminase; ALT, alanine transaminase; MELD-albumin, Model for End-stage Liver Disease with albumin replacing international normalized ratio. CPBT, cardiopulmonary bypass time.

Supplementary Table 3 univariable analysis in different groups based on MELD-albumin score

| Variables | MELD-albumin<8.58  (n=95) | MELD-albumin≥8.58  (n=57) | *P*  Value |
| --- | --- | --- | --- |
| Age, years | 54(50, 62) | 54(45, 66) | 0.828 |
| Female | 56(58.9) | 31(54.4) | 0.582 |
| BMI | 22.6±3.1 | 21.4±3.1 | **0.025^*^** |
| Smoking | 7(7.4) | 6(10.5) | 0.502 |
| Moderate drinking | 1(1.1) | 6(10.5) | **0.028^*^** |
| Hypertension | 11(11.6) | 6(10.5) | 0.842 |
| Stroke | 3(3.2) | 4(7.0) | 0.284 |
| Diabetes | 10(10.5) | 3(5.3) | 0.270 |
| NYHA class Ⅲ/Ⅳ | 57(60.0) | 39(68.4) | 0.298 |
| Atrial fibrillation | 60(63.2) | 32(56.1) | 0.392 |
| Previous cardiac surgery | 78(82.1) | 40(70.2) | 0.090 |
| Pacemaker implantation | 8(8.4) | 3(5.3) | 0.471 |
| Lower extremities edema | 42(44.2) | 35(61.4) | **0.041^*^** |
| Severe TR insufficiency | 91(95.8) | 54(94.7) | 0.765 |
| LVEF | 62.3±8.3 | 61.1±8.7 | 0.430 |
| Liver congestion | 42(44.2) | 26(45.6) | 0.866 |
| Pericardial effusion | 1(1.1) | 4(7.0) | 0.083 |
| Pleural effusion | 14(14.7) | 11(19.3) | 0.464 |
| AST | 26(22, 32) | 25(22, 32.5) | 0.654 |
| ALT | 18(14, 27) | 17(14, 23) | 0.432 |
| Albumin | 42(41, 44) | 37(34, 38) | **＜0.001^*^** |
| MELD score | 11.1±3.9 | 12.5±5.2 | 0.055 |
| MELD-XI score | 9.4(9.4, 9.4) | 9.4(9.4, 9.7) | **0.009^*^** |
| MELD-albumin score | 6.4(6.4, 7.3) | 10.2(9.4, 12.7) | **＜0.001^*^** |

^a^ BMI, body mass index; NYHA, New York Heart Association classification; TR, tricuspid regurgitation. LVEF, left ventricular ejection fraction; INR, international normalized ratio. AST, aspartate transaminase; ALT, alanine transaminase; MELD, Model for End-stage Liver Disease; MELD-XI, Model for End-stage Liver Disease excluding international normalized ratio; MELD-albumin, Model for End-stage Liver Disease with albumin replacing international normalized ratio.
