# Supplementary figures and images for: Prognostic Value of Modified Model for End-Stage Liver Disease Score in Patients Undergoing Isolated Tricuspid Valve Replacement
Source: Front Cardiovasc Med. 2022 Jul 1;9:932142. doi: 10.3389/fcvm.2022.932142 (PMC9283717; doi:10.3389/fcvm.2022.932142)

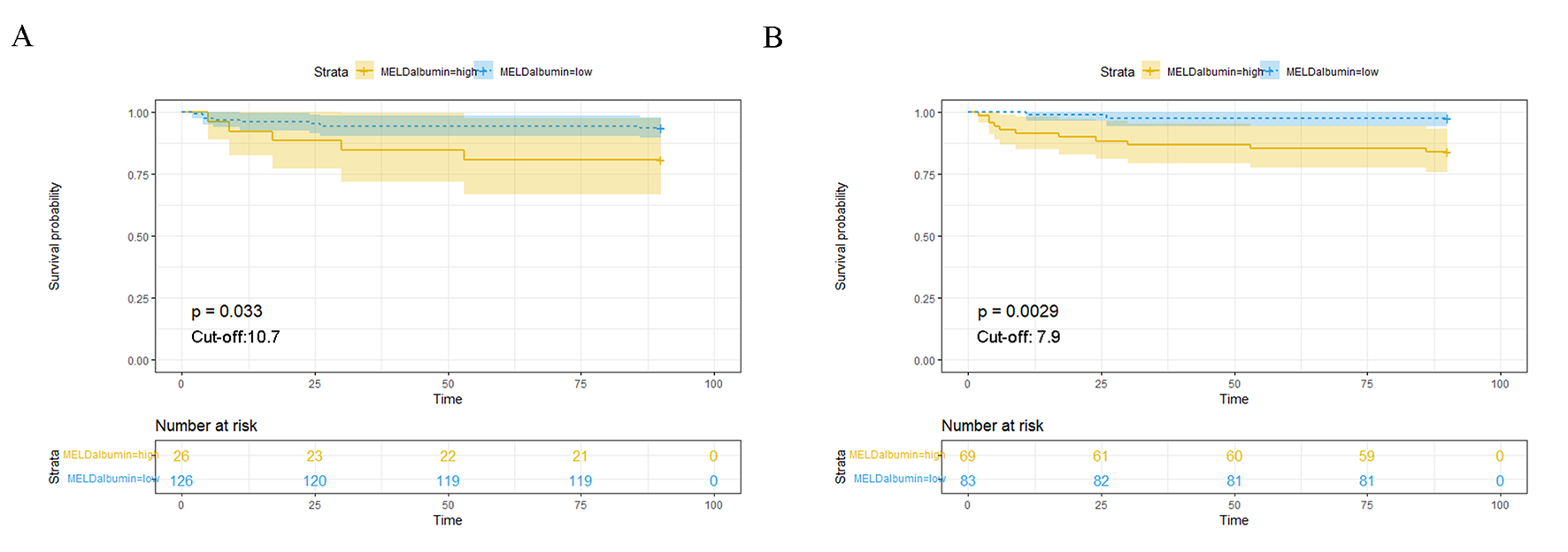

Supplement: Supplementary Figure 1 — Kaplan–Meier curve analysis for the in-hospital mortality with cutoff value of the model for end-stage liver disease (MELD)-albumin from previous studies. (A) Kaplan–Meier curve analysis for the in-hospital mortalitywith cutoff value of MELD-albumin as 10.7. (B) Kaplan–Meier curve analysis for the in hospitalmortality with cutoff value of MELD-albumin as 7.9. [file Image_1.TIF]
